# Short‐term outcomes in infants with mild neonatal encephalopathy: a retrospective, observational study

**DOI:** 10.1186/s12887-021-02688-y

**Published:** 2021-05-07

**Authors:** Yoshinori Aoki, Tatsuo Kono, Mikako Enokizono, Kaoru Okazaki

**Affiliations:** 1 Department of Neonatology, Tokyo Metropolitan Children’s Medical Center, 2-8-9 Musashidai, Fuchu, 183-8561 Tokyo, Japan; 2 Department of Radiology, Tokyo Metropolitan Children’s Medical Center, Tokyo, Japan

**Keywords:** Mild neonatal encephalopathy, Perinatal asphyxia, Magnetic resonance imaging, Brain, Thompson score, Outcome

## Abstract

**Background:**

Neonatal encephalopathy due to acute perinatal asphyxia is a major cause of perinatal brain damage. Moderate to severe neonatal encephalopathy is associated with high mortality and morbidity rates. However, the neurodevelopmental outcomes in neonates with mild neonatal encephalopathy are unclear. The primary aim of this single-center observational study was to assess the short-term outcomes in term neonates with mild neonatal encephalopathy due to perinatal asphyxia. A secondary aim was to identify predictors of poor prognosis by identifying the characteristics of these infants according to their short-term outcomes.

**Methods:**

We retrospectively investigated all infants with perinatal asphyxia at Tokyo Metropolitan Children’s Medical Center from January 2014 to December 2019. An abnormal short-term outcome was defined as any one of the following: seizures or abnormal electroencephalography, abnormal brain magnetic resonance imaging obtained within the first 4 weeks of life, and abnormal neurological examination findings at discharge.

**Results:**

In total, 110 term infants with perinatal asphyxia during the study period were screened and 61 were diagnosed with mild neonatal encephalopathy. Eleven (18 %) of these infants had an abnormal short-term outcome. The median Thompson score at admission was significantly higher in infants with abnormal short-term outcomes than in those with normal short-term outcomes (5 [interquartile range, 4-5.5] vs. 2 [interquartile range, 1–3], *p* < 0.01). Receiver operating characteristic curve analysis showed that a cutoff value of 4 had high sensitivity and specificity (90.9 and 83.0 %, respectively) for prediction of an abnormal short-term outcome.

**Conclusions:**

18 % of infants with mild encephalopathy had an abnormal short-term outcome, such as abnormal brain magnetic resonance imaging findings. The Thompson score at admission may be a useful predictor of an abnormal short-term outcome in infants with mild neonatal encephalopathy.

**Supplementary Information:**

The online version contains supplementary material available at 10.1186/s12887-021-02688-y.

## Background

Neonatal encephalopathy (NE, also known as hypoxic-ischemic encephalopathy) due to acute perinatal asphyxia is a major cause of perinatal brain damage [1–3]. NE has an incidence of 2 to 8 per 1,000 live births and is associated with high morbidity and mortality rates [4, 5]. Several randomized controlled trials have demonstrated the effectiveness of therapeutic hypothermia in infants with moderate or severe NE [6–8]. As a result, therapeutic hypothermia was described as a standard treatment for moderate and severe NE at the 2010 International Consensus Conference on Cardiopulmonary Resuscitation and Emergency Cardiovascular Care Science with Treatment Recommendations and is now used in many countries [9, 10].

On the other hand, infants with mild NE have been considered to have a good prognosis [11, 12]. Therefore, neuroprotective therapy such as therapeutic hypothermia is thought to be unnecessary for infants with mild NE. However, the neurological prognosis has been noted to be poor in some infants with mild NE [13–16]. Moreover, therapeutic hypothermia is often implemented for mild NE despite lack of sufficient evidence [17, 18].

In this study, we aimed to investigate the short-term outcomes in a single-center cohort of term neonates with mild NE. As a secondary aim, we sought to identify predictors of poor prognosis in these infants.

## Methods

### Study design

This single-center retrospective observational study assessed the short-term outcomes in term neonates with mild NE due to perinatal asphyxia at the Tokyo Metropolitan Children’s Medical Center between January 2014 and December 2019. This institution’s neonatal intensive care unit (NICU) is a tertiary center and provides medical care in the west of Tokyo, where about 30,000 infants are born annually. The study was approved by our institutional ethics committee. The need for informed consent was waived in view of the retrospective design of the study.

### Study population

All infants born during the study period were screened to identify those who were admitted to the NICU within 5.5 h of birth, were born at ≥ 36 weeks’ gestation, had a birth weight ≥ 1800 g, and showed evidence of perinatal asphyxia. Infants with major malformation or insufficient data were excluded. Newborns with perinatal asphyxia were identified by the same criteria as those used in a previous study of therapeutic hypothermia [7]: (1) pH < 7.0 or base deficit ≥ 16 mmol/L in a sample of umbilical cord blood or any blood during the first hour after birth; (2) an acute perinatal event with either a 10-min Apgar score ≤ 5 or assisted ventilation initiated at birth and continued for at least 10 min.

A standardized neurological examination was performed in newborns who met these criteria. The severity of NE was assessed using the modified Sarnat staging as in the National Institute of Child Health and Human Development  (NICHD) therapeutic hypothermia trial [7, 11]. Specifically, the severity of NE was classified based on the following 6 categories: level of consciousness, spontaneous activity, posture, tone, primitive reflexes (suck and Moro), and autonomic nervous system function (pupils, heart rate, or respiration). The parameters in each category were classified as normal, mild, moderate, or severe. Infants with a moderate or severe abnormality found in ≥ 3 categories were classified as having moderate or severe NE, based on the number of moderate or severe abnormalities. Infants with an abnormality (of any severity) in ≥ 1 category but no evidence of moderate or severe NE were classified as having mild NE. Infants with no abnormalities were classified as normal (without NE). We also evaluated the Thompson score on admission to the NICU. The Thompson score is a numeric scoring system that includes the following 9 neurological signs and vital parameters: tone, level of consciousness, fits (seizures), posture, Moro reflex, grasp reflex, suck reflex, fontanelle findings, and respiration. The total score ranges from 0 (normal) to 22 (severe) points [19].

Therapeutic hypothermia was performed for infants with moderate or severe NE without severe complications. Therapeutic hypothermia was administered in some infants with mild NE according to the discretion of the attending doctors.

### Outcomes

Abnormal short-term outcomes were defined as any one of the following at discharge from the NICU based on their relation with neurodevelopmental impairment: seizures or abnormal findings on electroencephalography [20, 21], abnormal brain magnetic resonance imaging (MRI) findings within 4 weeks of age [13, 17, 22, 23], abnormal neurological examination findings at discharge from the NICU [24].

Electroencephalographic and MRI studies were performed when clinically indicated. MRI protocols consisted of conventional T1-weighted, T2-weighted, and diffusion-weighted sequences. MRI scans were reviewed by experienced pediatric radiologists.

### Statistical analysis

Data are shown as the mean ± standard deviation for normally distributed continuous variables, as the median and interquartile range (IQR) for other continuous variables, and as the number and percentage of the total number of infants for categorical variables. Statistical analyses were performed using SPSS Statistics version 26.0 (IBM Corp., Armonk, NY). Differences between the groups were evaluated using the Mann-Whitney *U* test for continuous variables and Fisher’s exact test for categorical variables. The ability of the Thompson score to predict the short-term outcomes was assessed using receiver operating characteristic (ROC) curve analysis. A* p*-value < 0.05 was considered statistically significant.

## Results

**Fig. 1:**
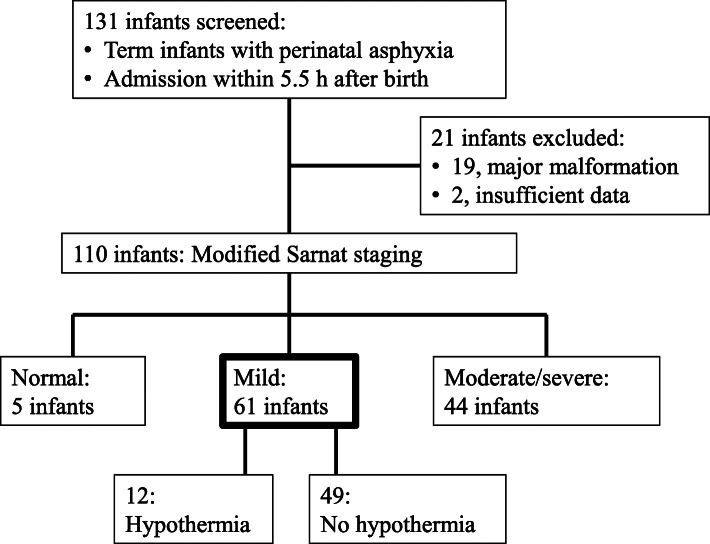
Enrollment, classification, and treatment of the study infants. Infants who met the inclusion criteria and none of the exclusion criteria. Perinatal asphyxia was defined as one or more of the following: 10 min Apgar score ≤ 5, resuscitation ≥ 10 min, blood gas pH < 7.0, and blood gas base deficit ≥ 16. One patient with Sarnat stage 2 neonatal encephalopathy did not receive therapeutic hypothermia due to severe bleeding

A total of 2883 infants were admitted to the NICU during the study period. Of these infants, 131 were identified to be term infants with perinatal asphyxia, 21 of which were excluded because of major malformations (n = 19) or insufficient data (n = 2). The remaining 110 eligible infants were categorized according to severity of NE (Fig. 1). Of these, 61 newborns were diagnosed to have mild NE and formed the study population. Five infants were diagnosed to be normal (without NE), 31 to have moderate NE, and 13 to have severe NE; the characteristics of these infants and their treatment course are shown in Additional file 1 Therapeutic hypothermia was provided for 12 infants (20 %) with mild NE according to the discretion of the attending doctors. The characteristics of infants who were treated with therapeutic hypothermia are shown in Additional File 2 At our facility, therapeutic hypothermia was more likely to be provided to infants who had a worse Apgar score at 5 min, had worse blood gas results, or required tracheal intubation and vasopressor. MRI studies were performed in 40 infants with mild NE as clinically indicated.

**Fig. 2:**
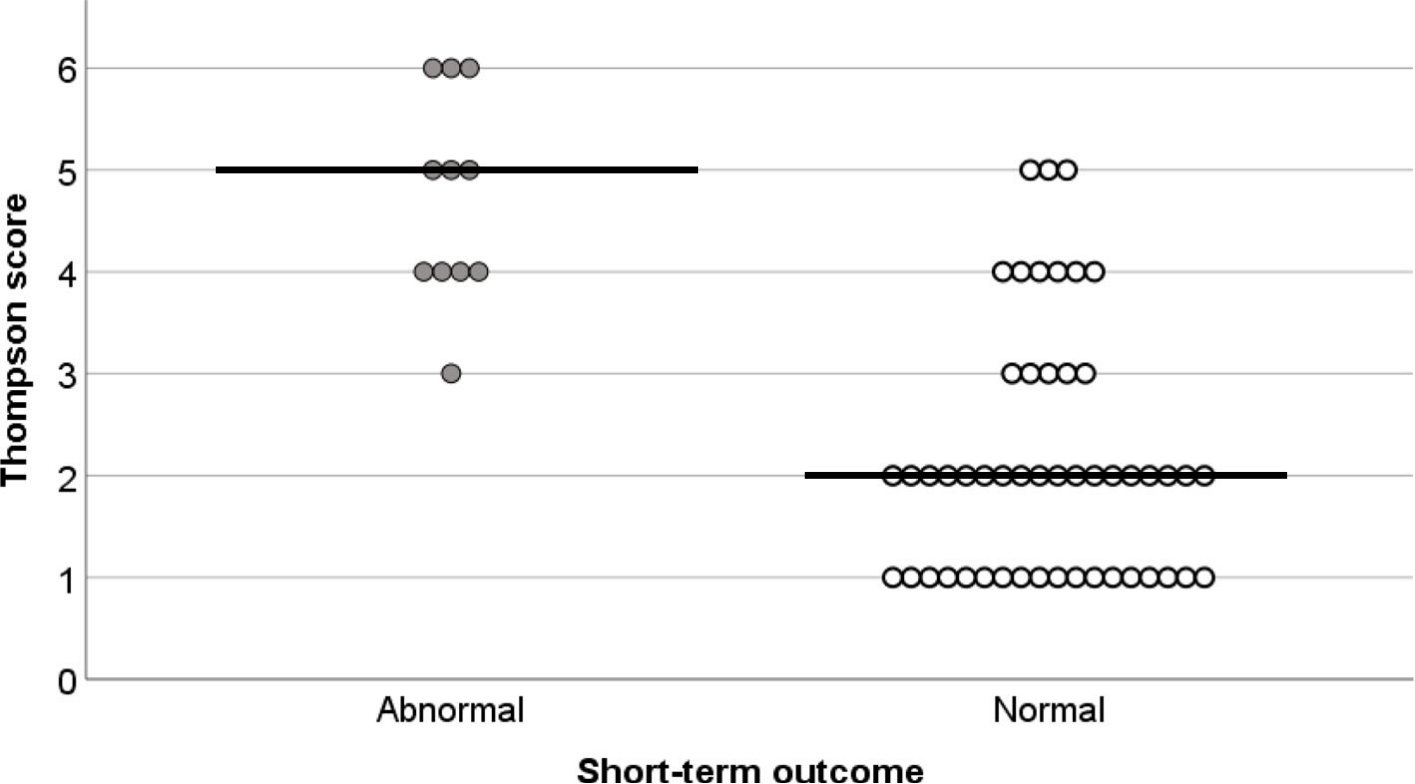
Thompson scores in infants with and without abnormal short-term outcomes. The horizontal line shows the median in the scatterplot. The scores were significantly higher in infants with abnormal short-term outcomes than in those with normal short-term outcomes (*p* < 0.01)

**Fig. 3:**
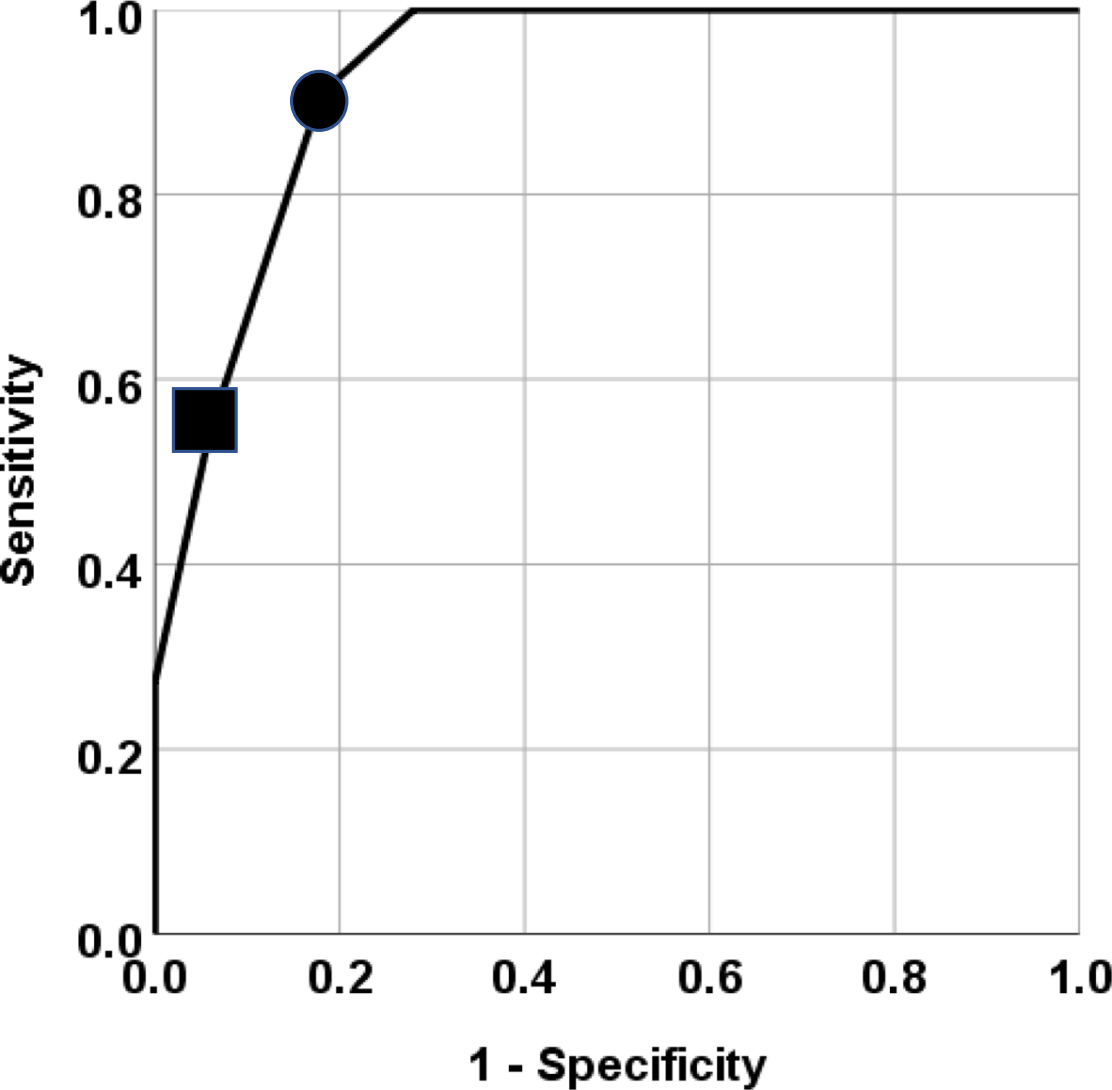
Receiver operating characteristic curve analysis of Thompson score as a predictor of short-term outcome. The area under the curve was 0.93 (95% confidence interval 0.86-0.99). The black circle and square indicate cutoff points of 4 and 5, respectively. When the cutoff value was 4, the sensitivity and specificity were 90.9% and 83.0%, respectively, and the positive and negative predictive values were 52.6% and 97.6%

Table 1 compares the infants with mild NE according to whether their short-term outcomes were normal or abnormal. Eleven infants (18 %) had some abnormalities at the time of discharge; 10 had abnormal brain MRI findings and 1 had abnormal findings on brain MRI, abnormalities on neurological examination at discharge, seizures, and abnormal background activity on electroencephalography. The abnormal brain MRI findings consisted of lesions in the basal ganglia and/or thalami in 6 infants, lesions in the white matter in 3 infants, lesions in the basal ganglia, thalami, and white matter bilaterally in 1 infant, and lesions in the cerebral cortex, white matter, basal ganglia, and thalami in the remaining infant. Further information on these infants is provided in Additional File 3. Some examples of abnormal MRI findings are shown in Additional File 4. There was no difference between the groups in maternal characteristics or complications, need for resuscitation in the delivery room, Apgar scores at 1 and 5 min, or the lowest blood gas pH value obtained in the first 6 h after birth (Table 1). The highest blood gas base deficit recorded within the first 6 h was significantly higher in the infants with abnormal short-term outcomes (median 17 [IQR 5–21] vs. 11 [IQR 7.8-19], *p* = 0.04). Furthermore, the Thompson scores at admission were significantly higher in infants with abnormal short-term outcomes than in those with normal short-term outcomes (median 5 [IQR 4-5.5] vs. 2 [IQR 1–3], *p *< 0.01; Fig. 2). ROC curve analysis of the ability of the Thompson score at admission to predict an abnormal short-term outcome revealed an area under the curve of 0.93 (95 % confidence interval, 0.86–0.99; Fig. 3). When the cutoff value was 4, the sensitivity and specificity were 90.9 and 83.0 %, respectively, with positive and negative predictive values of 52.6 and 97.6 % (Table 2). We also assessed the effects of therapeutic hypothermia. One (8 %) of the 12 infants with an abnormal short-term outcome had received therapeutic hypothermia. In contrast, 11 (22 %) of the 49 infants who did not have any abnormal neurological findings at discharge had received therapeutic hypothermia (22 %). However, there was no significant difference (*p* = 0.33).

**Table 1:** Comparison between infants with mild NE according to whether short-term outcomes were normal or abnormal

	Abnormal	Normal	*p*-value
Infants, n (%)	11	50	
Male sex, n (%)	6 (55)	32 (64)	0.56
Gestational age (weeks), median (IQR)	39 (39–39)	39 (37.5–40)	0.93
Birth weight (g), mean ± SD	2848 ± 378	2934 ± 529	0.48
Mode of delivery			
Cesarean delivery, n (%)	4 (36)	22 (44)	0.64
Vacuum extraction, n (%)	3 (27)	9 (18)	0.48
Natural, n (%)	4 (36)	19 (38)	0.89
Non-reassuring fetal status, n (%)	10 (91)	28 (56)	0.09
Placental abruption, n (%)	3 (27)	11 (22)	0.59
Outborn delivery, n (%)	7 (64)	43 (86)	0.09
Apgar score at 1 min, median (IQR)	2 (1-2.5)	2 (1–3)	0.14
Apgar score at 5 min, median (IQR)	5 (3-5.5)	5 (4–6)	0.38
Intubation (in delivery room), n (%)	6 (55)	32 (64)	0.56
Cardiac compression, n (%)	2 (18)	2 (4)	0.09
CPR ≥ 10 min, n (%)	9 (82)	41 (82)	1.0
Blood gas pH, median (IQR)	6.97 (6.89–7.11)	7.03 (6.92–7.15)	0.69
Blood gas base deficit, median (IQR)	17 (15–21)	11 (7.8–19)	0.04
Thompson score, median (IQR)	5 (4-5.5)	2 (1–3)	< 0.01
Vasopressor use, n (%)	2 (18)	15 (30)	0.43
Therapeutic hypothermia, n (%)	1 (9)	11 (22)	0.33

Blood gas pH: lowest pH in either umbilical cord blood or infant blood recorded within the first 6 h of birth. Blood gas base deficit: highest base deficit in either umbilical cord blood or infant blood recorded within the first 6 h of birth. *CPR* cardiopulmonary resuscitation; *IQR* interquartile range; *SD* standard deviation

**Table 2:** Sensitivity, specificity, PPV, and NPV of the Thompson score in prediction of abnormal short-term outcomes

Thompson score	Sensitivity	Specificity	PPV	NPV
2	100 %	36 %	25 %	100 %
3	100 %	73 %	44 %	100 %
**4**	**90.90 %**	**83 %**	**52.60 %**	**97.60 %**
5	54.50 %	94 %	66.70 %	90.40 %
6	27.30 %	100 %	100 %	86.20 %

*NPV* negative predictive value; *PPV* positive predictive value

A Thompson score of 4 (shown in bold) had high sensitivity and specificity (90.9 and 83.0 %, respectively) with positive and negative predictive values of 52.6 and 97.6 %.

## Discussion

In this study, we found that 18 % of infants with mild NE had abnormal short-term outcomes such as abnormal brain MRI findings, abnormal neurological examination findings at discharge, or seizures. The Thompson score at admission may be useful for prediction of abnormal short-term outcomes in infants with mild NE.

Unlike moderate or severe cases, infants with mild NE have been considered to have a good prognosis [11, 12]. However, accumulating evidence suggests that neonates with mild NE are also at risk for neurodevelopmental disorders [13–16]. One reason for these inconsistent reports may be a difference in timing of evaluation, in that the earlier studies defined mild NE by serial neurological examination during the first week after birth whereas the therapeutic hypothermia trials defined the severity of NE based on evaluation within 6 h of birth [25]. Moreover, the timing and methods used to assess the prognosis varied in the earlier studies. In the present study, we found that a considerable number of infants with mild NE had abnormal short-term outcomes, which is consistent with previous studies. Most of the infants with abnormal short-term outcomes in this study had abnormal brain MRI findings; furthermore, there may have been infants with poor prognosis who went undetected because brain MRI was not performed in all cases. Although we did not assess neurodevelopmental outcomes at older ages, brain MRI abnormalities obtained within 4 weeks after birth are reported to be associated with neurodevelopmental impairments [22, 23]. In addition, we must consider not only problems that occur in early childhood but also those that arise in school-aged and adolescent children. van Kooij et al. reported that MRI abnormalities found in the neonatal period are also related to intelligence quotient (IQ) scores obtained in school-aged children and in children with special educational needs, and that even among infants with normal or mild lesions on neonatal MRI, 23.1 % (3/13) had an IQ score ≤ 85 [26]. Furthermore, neuropsychological problems, such as poor attention/executive function, poor memory performance, and behavioral difficulties, have been identified in studies that have followed children with NE through to school age [27]. The long-term neuropsychological outcome of mild NE is controversial; some papers report that even mild NE can cause psychological problems [28, 29] while others report no concern if they are not severe [30]. Although the long-term prognosis of mild NE is not fully understood, we should be aware that the risk of poor neurological prognosis in children with NE of mild severity is not negligible. Therefore, it is important to continue to follow up infants with mild NE in the long term and accumulate data on their long-term outcomes.

Although the Sarnat score is the method most widely used to test for NE, the Thompson score at admission may also be a useful predictor of the short-term outcome in infants with mild NE. Introduced by Sarnat et al. in 1976, the Sarnat score requires evaluation of many items and is somewhat complicated to use [11]. The Thompson score was originally introduced as a simple and effective scoring system for predicting neurodevelopmental outcomes in neonates with hypoxic-ischemic encephalopathy before the era of therapeutic hypothermia. It is a numeric scoring system that requires no equipment or specific training to use. Scores ≥ 15 have been associated with cerebral palsy or severe developmental retardation [19]. Another study reported that the median Thompson score was 4 in mild to moderate encephalopathy and 9 in severe encephalopathy, which is consistent with our data for mild encephalopathy [31]. The Thompson score also has a high predictive value for outcomes in infants treated with therapeutic hypothermia [32]. Although we found that a Thompson score of 4 was a highly sensitive and highly specific predictor of an abnormal short-term outcome, the value was lower than that used by earlier researchers. Compared with previous studies of the predictive ability of the Thompson score [19, 32], we set a milder definition of abnormal outcomes. Therefore, it is possible that our cutoff score was lower than that in previous studies. A recent prospective study by Chalak et al. found that the total Sarnat score recorded in the first 6 h after birth can predict disability at 18–22 months in infants with mild NE [33]. We propose inclusion of this simple scoring system, the Thompson score, into the prognostic assessment of mild NE, which is often treated in institutions without a neonatal neurological specialist.

We could not reach a conclusion regarding the effectiveness of therapeutic hypothermia for mild NE. While there are some papers suggesting that therapeutic hypothermia is effective in mild NE [34, 35], a recent meta-analysis could not confirm its effectiveness [36]. Although therapeutic hypothermia is often already provided for infants with mild NE, prospective trials are needed to confirm its efficacy in these infants. Our present findings suggest that poor prognosis is unlikely in children with milder NE who have a lower Thompson score, such as 1 or 2, but that it is necessary to screen high-risk infants with mild NE in the trials. We hope that our findings will be useful in future research.

The limitations of this study include its retrospective design and lack of long-term follow-up in most cases. However, some infants had brain MRI abnormalities at discharge, which is associated with poor long-term prognosis. Therefore, we assume that mild encephalopathy does not always have a good prognosis.

## Conclusions

We found that the neurological prognosis can be poor even in mild NE. The Thompson score at admission may be useful for prediction of abnormal short-term outcomes in infants with mild NE. Our findings may be helpful for selection of high-risk infants for enrolment in future trials of neuroprotective therapy.

## Supplementary Information

Additional file 1. Maternal and neonatal characteristics and short-term outcomes in infants. This file shows maternal and neonatal characteristics and short-term outcomes of all screened infants by severity.

Additional file 2. Comparison among infants with mild NE according to whether therapeutic hypothermia was provided or not. This file shows maternal and neonatal characteristics of infants with mild NE according to whether therapeutic hypothermia was provided or not.

Additional file 3. Details of infants with abnormal short-term outcomes. This file shows detailed data for infants with mild neonatal encephalopathy who had an abnormal short-term outcome.

Additional file 4. Examples of abnormal MRI findings. A. Axial T_1_-weighted sequence at 6 days of age showing a focal high signal intensity lesion of the white matter (Patient No. 2). B. Axial T_1_-weighted sequence at 2 days of age showing bilateral high signal intensity lesions in the globus pallidus and subthalamic nuclei (Patient No. 5). C. Axial T_1_-weighted sequence at 25 days of age showing bilateral high signal intensity changes and atrophy in the basal ganglia and thalamus as well as multicystic encephalomalacia in the greater part of the bilateral cerebral hemispheres (Patient No. 8).

## Data Availability

The datasets used and analyzed during the current study are available from the corresponding author on reasonable request.

## Abbreviations

CPR: Cardiopulmonary resuscitation
IQ: Intelligence quotient
IQR: Interquartile range
MRI: Magnetic resonance imaging
NE: Neonatal encephalopathy
NICU: Neonatal intensive care unit
NPV: Negative predictive value
PPV: Positive predictive value
ROC: Receiver operating characteristic
