# Supplementary material for: Short‐term outcomes in infants with mild neonatal encephalopathy: a retrospective, observational study
Source: BMC Pediatr. 2021 May 7;21:224. doi: 10.1186/s12887-021-02688-y (PMC8103637; doi:10.1186/s12887-021-02688-y)
Supplement: Supplementary file 4 — Examples of abnormal MRI findings. A. Axial T1-weighted sequence at 6 days of age showing a focal high signal intensity lesion of the white matter (Patient No. 2). B. Axial T1-weighted sequence at 2 days of age showing bilateral high signal intensity lesions in the globus pallidus and subthalamic nuclei (Patient No. 5). C. Axial T1-weighted sequence at 25 days of age showing bilateral high signal intensity changes and atrophy in the basal ganglia and thalamus as well as multicystic encephalomalacia in the greater part of the bilateral cerebral hemispheres (Patient No. 8). [file 12887_2021_2688_MOESM4_ESM.pptx]

## Slide 1
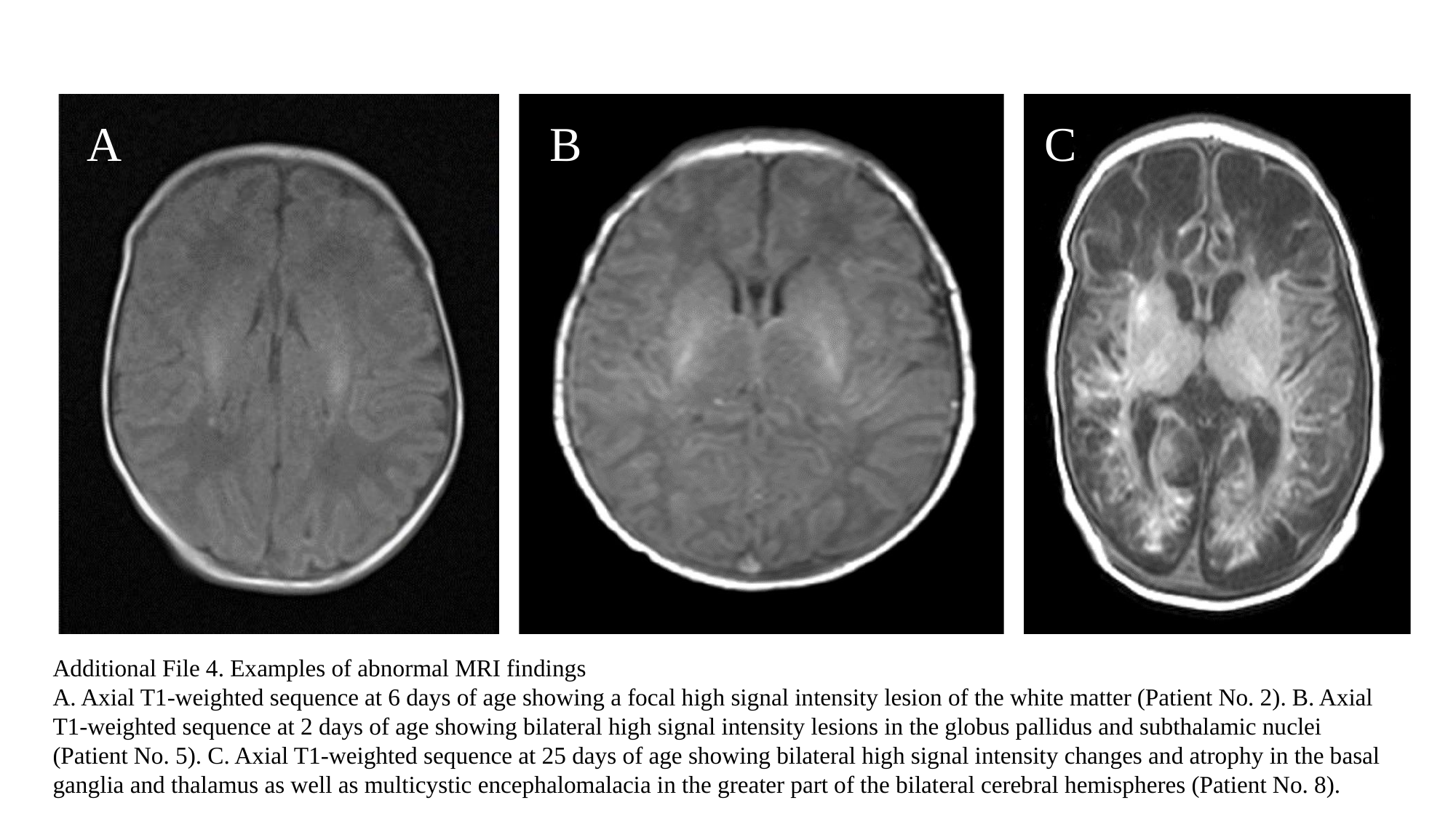

A
B
C
Additional File 4. Examples of abnormal MRI findings
A. Axial T1-weighted sequence at 6 days of age showing a focal high signal intensity lesion of the white matter (Patient No. 2). B. Axial T1-weighted sequence at 2 days of age showing bilateral high signal intensity lesions in the globus pallidus and subthalamic nuclei (Patient No. 5). C. Axial T1-weighted sequence at 25 days of age showing bilateral high signal intensity changes and atrophy in the basal ganglia and thalamus as well as multicystic encephalomalacia in the greater part of the bilateral cerebral hemispheres (Patient No. 8).
